# A Case

**Published:** 1887-05

**Authors:** 


**A Case** illustrative of maternal influence on the fœtus late in pregnancy is reported by Dr. W. J. Swift in a late number of the *New York Medical Journal.* The mother was much grieved on account of an injury resulting in a severe contusion of the thumb nail of her little boy who was about two years old. Three weeks later her baby was born, and its right thumb presented an appearance identical with that of its brother's wounded thumb.—*Practice.*

